# Collective Intelligence: Aggregation of Information from Neighbors in a Guessing Game

**DOI:** 10.1371/journal.pone.0153586

**Published:** 2016-04-19

**Authors:** Toni Pérez, Jordi Zamora, Víctor M. Eguíluz

**Affiliations:** Instituto de Física Interdisciplinar y Sistemas Complejos IFISC (CSIC-UIB), E07122 Palma de Mallorca, Spain; University of Sheffield, UNITED KINGDOM

## Abstract

Complex systems show the capacity to aggregate information and to display coordinated activity. In the case of social systems the interaction of different individuals leads to the emergence of norms, trends in political positions, opinions, cultural traits, and even scientific progress. Examples of collective behavior can be observed in activities like the Wikipedia and Linux, where individuals aggregate their knowledge for the benefit of the community, and citizen science, where the potential of collectives to solve complex problems is exploited. Here, we conducted an online experiment to investigate the performance of a collective when solving a guessing problem in which each actor is endowed with partial information and placed as the nodes of an interaction network. We measure the performance of the collective in terms of the temporal evolution of the accuracy, finding no statistical difference in the performance for two classes of networks, regular lattices and random networks. We also determine that a Bayesian description captures the behavior pattern the individuals follow in aggregating information from neighbors to make decisions. In comparison with other simple decision models, the strategy followed by the players reveals a suboptimal performance of the collective. Our contribution provides the basis for the micro-macro connection between individual based descriptions and collective phenomena.

## Introduction

Decision making nowadays is a topic of growing interest from the scientific community and the industry. This is because, it can provide statistical predictions of animal and human behavior [1–6]. The effects of social influence and collaboration on the collective outcome have been addressed from different perspectives, both theoretical and experimental [7–11]. In complex problem solving, crowd-sourced collaboration has proved to be beneficial by shortening substantially the time to find a solution. Examples of these collaborative initiatives include problem solving in mathematics [12], software design [13], and data analysis [14].

In the study of cooperation, network reciprocity is an important mechanism [15, 16]. Theoretical works have shown that the role of network connectivity on cooperation depends on the evaluation of the individuals payoffs that can have a positive effect [17, 18], however, cooperation can be diminished when the real connection cost is taken into account [19]. Experimental work in the Prisoner’s dilemma game shows that heterogeneous networks do not enhance cooperation [20]. However, a dynamic interaction network can promote cooperation [21, 22]. Other works have also addressed how individuals learn and how the interaction network can influence the dissemination of useful information, individual choices, and the social outcomes [23, 24]. The performance of groups when compared with the performance of individual experts is reflected in The Wisdom of Crowds effect [25, 26] which suggests that the aggregation of independent decisions often outperforms individual experts. However, social influence can have a diminishing effect over this phenomenon [27].

To investigate how network structure affects social information use, decision making and decision accuracy, we performed an experiment with neutral items where individuals have to assemble information from peers according to an interaction network. Players have to make decisions based on incomplete and uncertain information, which allow us to address the process of aggregation of information and assess the decision making capacities of the players.

## Materials and Methods

### Ethics Statement

This study was approved by the Ethics Committee of the University of the Balearic Islands. Online informed consent was obtained from each participant prior to participation in the experiment.

### Experimental Design

**Fig 1:**
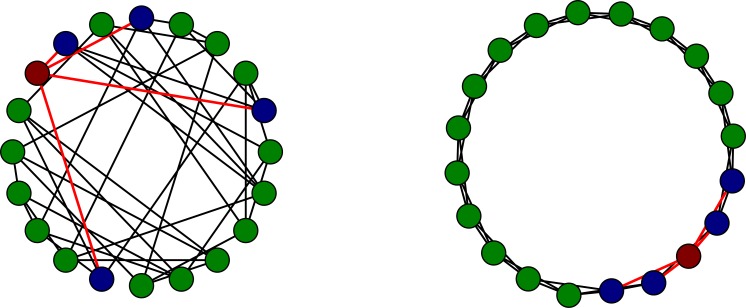
Illustration of the network configurations. Focal player (red node) is connected to k = 4 neighbors (blue nodes) having access to their proposals, and, at the same time, she shares her proposals with them. The remaining nodes and network connections are depicted by the green nodes and the gray links, respectively. Random network is represented on the left and regular network on the right.

We developed a social experiment consisting of an online game in which players have to guess a sequence of colors using information of an incomplete sequence provided initially to them and from the proposals of their neighbors. The experiment was structured in sessions each consisting of a set of *N* individuals assigned randomly to the nodes of a network as sketched in Fig 1. The target color code was composed of a sequence of *l*_*i*_ positions (*i* = 1, …, 10) colored with color *c*(*i*) from the available set (red, blue, and yellow). We defined *x*_*j*_(*l*_*i*_, *t*) as the color chosen by player *j* for the position *l*_*i*_ at time *t*.

We conducted a series of online sessions on different dates. The first one took place on May 7th, 2014 at 12:30 GMT+1 (Experiment 1) with 20 participants and a second experiment was conducted 3 weeks later on May 28th, 2014 at 11:30 GMT+1 (Experiment 2) with 17 participants. Both experiments were announced through a mailing list, and the players’ participation was voluntary and anonymous. Prior to the start of the experiment, the participants were required to agree on the Terms and Conditions and they were invited to answer a survey providing basic demographic data. The participants, 74% males and 26% females, reported an average age of 33.6 ± 7.3 years.

Each experiment consisted of six consecutive games being the first game intended to familiarize the participant with the interface and the purpose of the last game to evaluate the adaptation process of the players. The first and the last games were played against automata. In the remaining games, the participants interacted with other participants. Players were not informed whether they played with automata or with human players. An itemized list of instructions was provided at the beginning of each experiment. During the game, the participants also had access to a summary of the main instructions available on the screen (see S1 Text for a description of the instructions provided). No economical incentive was offered to the participants.

**Fig 2:**
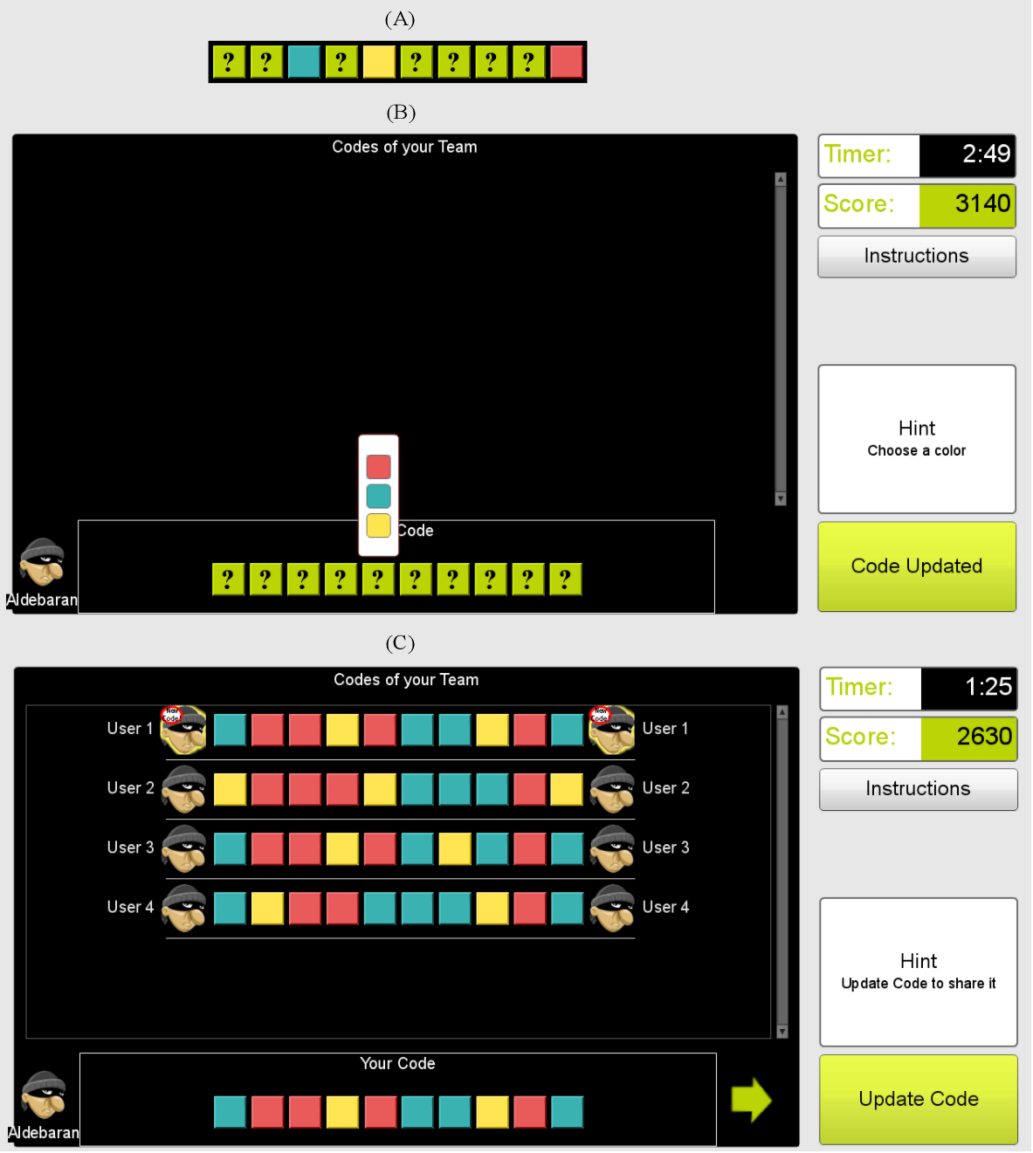
Interactive interface of the game. (A) Example of the partial target code shown initially to the player displaying positions 3, 5 and 10 with the correct color, while the other positions are empty. Green boxes with question marks are the remaining positions of the code to be guessed by the player (green color is not used in the color code sequence). (B) Interface for the first guess. When the mouse cursor is placed over a question mark green box, a list of available colors present in the code is shown. (C) Player interface during the game. At the top of the screen the proposals from neighboring players (Team) are shown. When a member of the Team updates her code, a flashing ‘new code’ bubble is shown on the icon of the updater.

In each game, players had to guess a color code of ten positions. Each position could be colored with one of the following colors: red, blue, or yellow. Participants had 225 seconds to guess the code after initial access to a partial sequence of the code. An example as well as the proposed codes of the neighboring players is shown in Fig 2A.

At the beginning of the game, only three out of the ten positions of the common target color code were shown to the players. The positions shown were generated at random covering the full code and they were randomly assigned to the players. After seeing the initial partial code, the players faced an empty code (green boxes with question marks) that they had to fill with their proposals. When the cursor was placed over one of the positions, a list of available colors was displayed on top of it (see Fig 2B). To avoid any bias towards any particular color, the order of the list of available colors presented to each player was randomly generated. When a player choses one of the colors in the list, the position is colored accordingly. Once the player fillS the entire color code, the update button is activated to submit and share the proposed color code. The player could then see the proposals of the neighbors only after the completion of the first guess.

Each player was initially randomly assigned to a node in the network and was limited to share her proposals only with the neighbors. Consequently, players only saw the proposals of their neighbors. Fig 2C shows the game interface after the first proposal of the focal player now including the proposals of the neighbors. The visualization interface limits the size of the neighborhood, and, to simplify the analysis, we considered networks where each node had the same number of neighbors. In particular we used a regular lattice and a degree regular random network as they show differentiated topological properties, for instance, with respect to the average path length. In order to avoid the effect of learning, if any, the topological arrangement of the networks in Experiment 1 was random, regular, regular, and random, while in Experiment 2 it was permuted to regular, random, random, and regular. In all network configurations, the node degree was set to *k* = 4.

At the end of each game, the players were rewarded with 10 points for finishing the game, and 100 points per correct position. A bonus of 1000 points was granted to the players guessing the correct code. Before starting a new game, a ranking of the players was shown. This was shown as an incentive to the players although but it was not used in the analysis. A movie with the dynamics of one of the games can be found in the Supporting Information (S1 Video).

## Results

**Fig 3:**
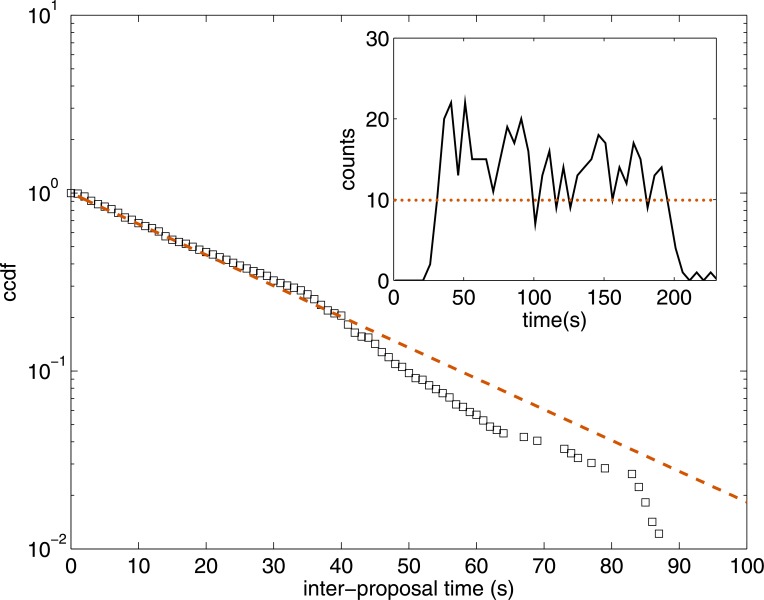
Activity of the players during the games. **Main figure**: complementary cumulative distribution function (ccdf) of the inter-proposal times (bin size: 1 s). Dashed line shows an exponential fit (MLE) to the data *f*(*t*) = exp(−*bt*) with *b* = 0.036 s^−1^. **Inset**: Temporal distribution of the number of proposals across the games aggregated in bins of 5 s. Dotted line shows the averaged activity of 10 proposals every 5 seconds.

The activity during the game is measured by the number of complete color codes submitted by the players. Fig 3 shows how the activity of the players during the games fluctuates around an average activity of 10 proposals every 5 seconds. The complementary cumulative distribution of inter-proposal time, that is, the time between two consecutive proposals for the same individual, reveals an exponential decay of the individuals’ activity with a characteristic time of 28 ± 3 s after Maximum Likelihood Estimation (MLE) [28].

**Fig 4:**
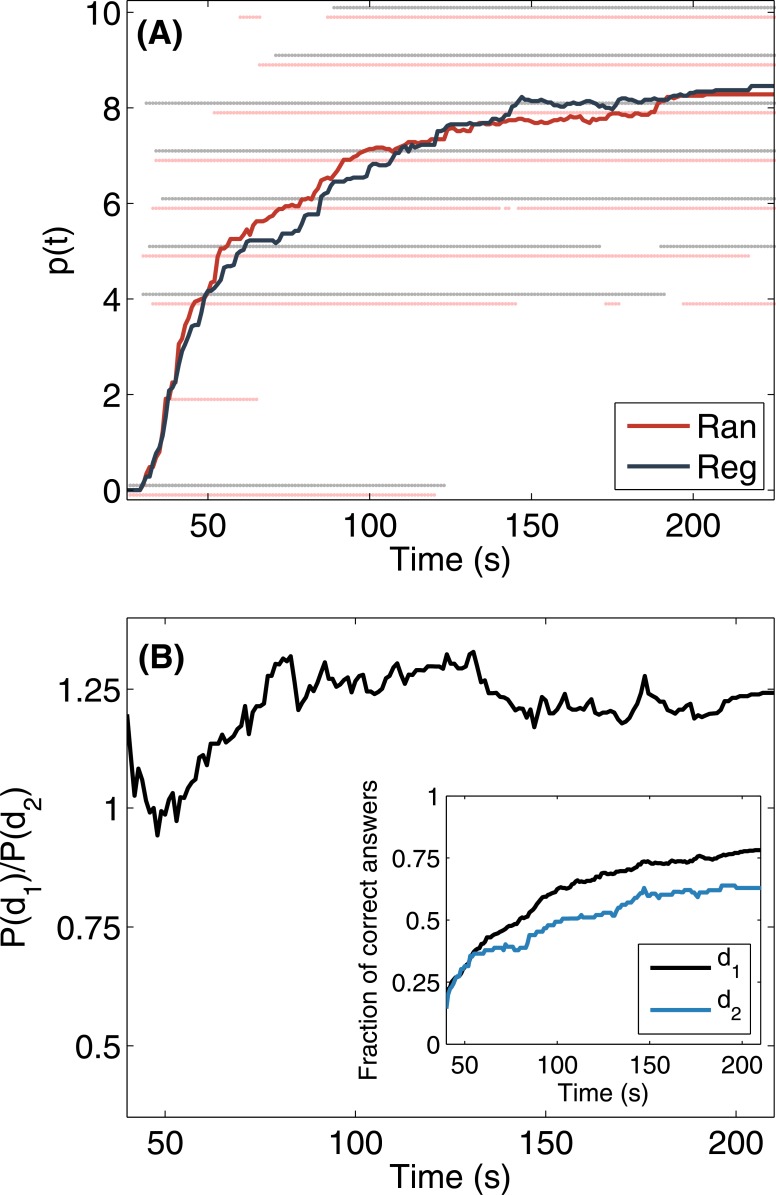
Group performance: temporal evolution of the accuracy. **(A)** Average number of correct positions for aggregated data of the games with the same interaction topology (solid thick lines, red random networks; blue: regular lattice). The number of correct positions of the individual players is also shown (dimmed symbols). For visualization purposes a slight vertical displacement has been applied. **(B)** Ratio between the probabilities of correct positions at distances *d*_1_ = 1 and *d*_2_ = 2 from the closest source. **Inset**: Temporal evolution of the fraction of correct positions for distances *d*_1_ and *d*_2_ to the closest source. In both panels, results are aggregated over all the sessions of the experiments.

In all the games, as the time increases, the participants tend toward the proposed color code. The hamming distance, defined as the number of differing positions between two color codes, averaged over all pairs of players at the end of each game lies in the range (2.1, 3.3). The performance of the collective is measured by the temporal evolution of the accuracy averaged over the total number of players, that is, $p\left(t\right)=\frac{1}{N}\sum_{j}\sum_{i}\delta_{x_{j}\left(l_{i},t\right),c\left(i\right)}$, where *δ*_*a*, *b*_ is the Kronecker’s delta. Fig 4A shows *p*(*t*) as well as the individual distributions for the two networks considered in the experiment. The average performance of the group at the end of the games with the same interaction network is *p* = 8.3. The unpaired Mann-Whitney U test and the paired Wilcoxon signed rank test, computed for the distributions of *p*(*t*) values at each time step of 1 s indicate the absence of any statistical difference between the distributions of *p*(*t*) in the two networks (average p-values > 0.4, see S1 Fig).

The propagation of information is investigated by measuring the relationship between the probability of providing a correct answer and the distance to the closest source. For a position, a node is a source if the position corresponds to one of the entries provided initially to the player in that node. The positions shown initially are correctly assigned 95% of the times by the players and practically remain unchanged during the game, with only 26 changes for these positions out of the 1491 proposals. For each player and each unknown position *l*_*i*_, we compute the shortest distance to the closest source and the number of correct answers at a given distance *d*_*i*_. For the two topologies and the experimental conditions, any player and any unknown position is at maximum distance of two to the closest source. The ratio between the probabilities of correct answers given at distance 1 and 2 from the sources indicates that proximity to source provides an advantage (24.4% on the average) to get the correct color (Fig 4B).

The adaptation of the players across the first and the last game in each experimental session is quantified by the averaged inter-proposal time. We observe similar values for the two experiments, and although the average values are similar, the last automata game shows systematically a smaller average inter-proposal time (S2 Fig).

### Transition events and probabilities

**Fig 5:**
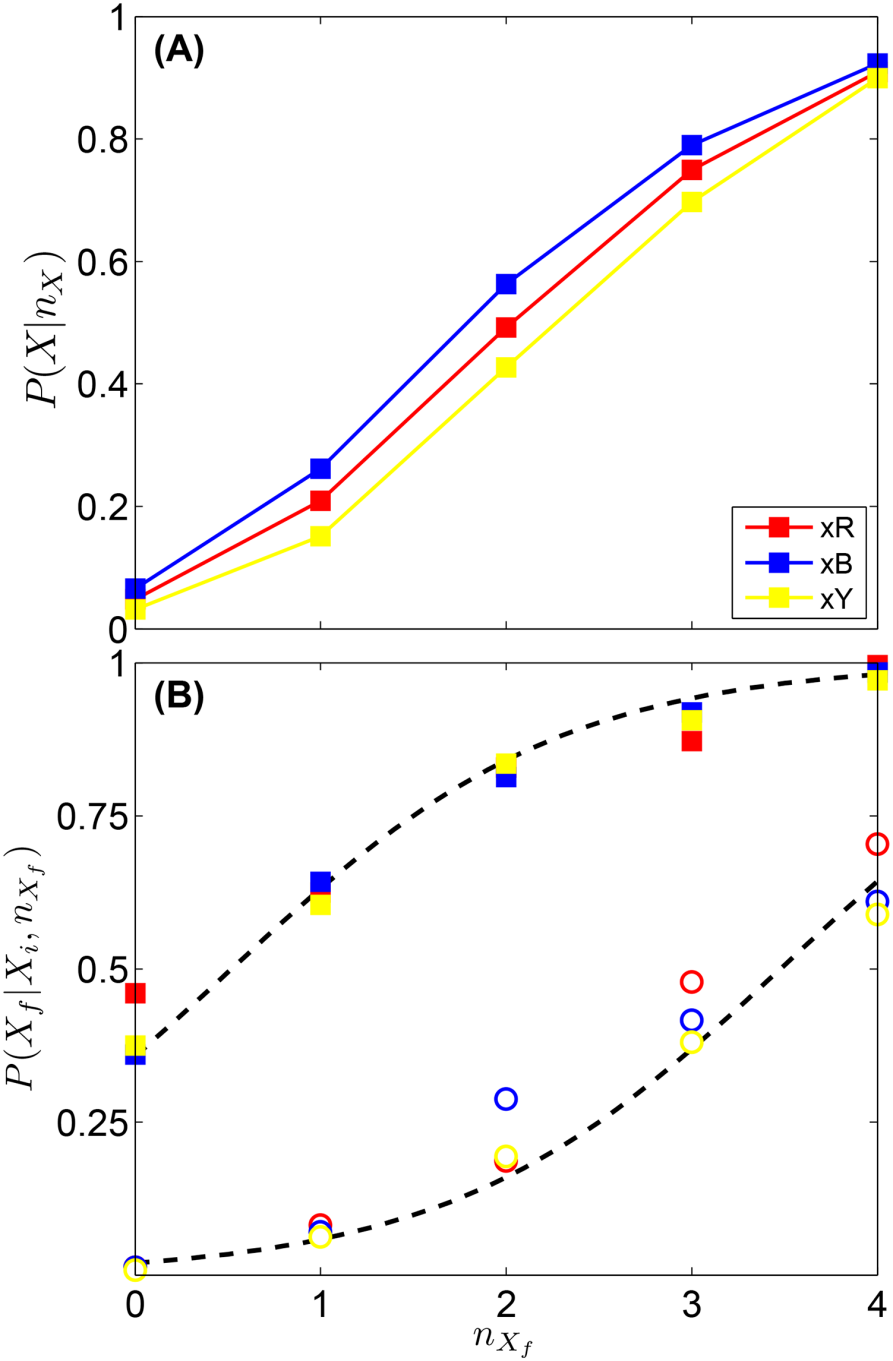
Transition Probabilities. **(A)** Probability *P*(*X*|*n*_*X*_) to change the unknown positions of the code from any color (R, B or Y) to a given color *X* (R, B or Y) as a function of *n*_*X*_, this is, the number of nearest neighbors already with color *X*. Results for each color are represented by the corresponding colored symbols. **(B)** Probabilities *P*(*X*_*f*_|*X*_*i*_; *n*_*X*_*f*__) for the unknown positions of the code as a function of *n*_*X*_*f*__. Open and solid symbols represent, for a given position, transitions between different colors ($\bar{X}X$) and between the same color (*XX*) respectively. Results for each color are represented by the corresponding colored symbols. Dashed lines represent fitting Eq (5) to the data (see Section Bayesian update for more details).

Given the sequence of proposals of one player and the color codes of the neighbors, we calculate the transition probabilities between different colors. We consider that each position is independent of each other, thus, we average the transition probabilities over all positions. From all the possible transitions between colors, we compute the conditional probability to change from any color to a given color *X*, *P*(*X*|*n*_*X*_) given *n*_*X*_ neighbors in state *X*. This probability, shown in Fig 5A, indicates that colors are practically equivalent for the players. This probability also points that, for a given position, as the number of neighbors with the same color *n*_*X*_ increases, the higher the probability that the player will switch to that color (or remain on it).

In addition, the probability to change to a different color $P(X|\bar{X},n_{X})$ and the probability to remain in the same color *P*(*X*|*X*, *n*_*X*_) measure the influence of the neighborhood to either transitions between colors $X\bar{X}$ or to remain in the same color (transitions *XX*). As Fig 5B shows, both the probability to change to color *X*_*f*_ and the probability to stick with color *X*_*f*_ increase with the number of neighbors *n*_*X*_*f*__ having color *X*_*f*_. The neighborhood provides stronger confirmation of the current state of the individual than exerts pressure to change it. This is, given a neighborhood configuration with *n*_*X*_*f*__, the probability for a player to stay in her current state (being *X*_*f*_) is greater than the probability of changing her state (being not *X*_*f*_) to *X*_*f*_. Interestingly, the conviction of a player in her current state can be quantified by the value of *P*(*X*|*X*, 0) ≈ 0.4, which matches $P(\bar{X}|X,n^{*})$ for n* = 3, corresponding to the neighborhood majority since the degree of the interaction networks was set to *k* = 4.

### Bayesian update

We assume players update their beliefs based on their previous belief and the information observed from the neighborhood. Then, following Bayes theorem, the posterior belief of agent *i* in state *X* = *R*, *B*, *Y* conditioned to receiving a signal *A* = *R*, *B*, *Y* is updated as follows,

$$\begin{array}{ccc} P_{post}\left(X|A\right) & = & \frac{P\left(A|X\right)P_{pre}\left(X|A\right)}{\sum_{x_{i}=R,B,Y}P\left(A|x_{i}\right)P_{pre}\left(x_{i}|A\right)}. \end{array}$$ (1)

By iterating Eq (1), the posterior belief of player *i* after receiving *n*_*R*_ red, *n*_*B*_ blue, and *n*_*Y*_ yellow signals is

$$\begin{array}{ccc} P_{post}\left(X|A\right) & = & \frac{P{\left(R|X\right)}^{n_{R}}P{\left(B|X\right)}^{n_{B}}P{\left(Y|X\right)}^{n_{Y}}P_{pre}\left(X|A\right)}{\sum_{x_{i}=R,B,Y}P{\left(R|x_{i}\right)}^{n_{R}}P{\left(B|x_{i}\right)}^{n_{B}}P{\left(Y|x_{i}\right)}^{n_{Y}}P_{pre}\left(x_{i}|A\right)}. \end{array}$$ (2)

Note that $\sum_{A_{i}=R,B,Y}P_{post}\left(X|A_{i}\right)=1$. Now, assuming that *P*(*A*|*X*) = *C*_0_ for *A* = *X* and *P*(*A*|*X*) = *C*′ for *A* ≠ *X*, Eq (2) becomes

$$\begin{array}{ccc} P_{post}\left(X|A\right) & = & \frac{1}{1+\sum_{x_{i}\neq X}{\left(\frac{C^{\prime }}{C_{0}}\right)}^{n_{X}-n_{x_{i}}}\frac{P_{pre}\left(x_{i}|A\right)}{P_{pre}\left(X|A\right)}}. \end{array}$$ (3)

The first hypothesis to consider is that the previous belief of the players, *P*_*pre*_(*x*_*i*_|*A*), is not related to the actual state of the player and it takes the same value for the different states *X*. In this case, Eq (3) reduces to

$$\begin{array}{ccc} P_{post}\left(X|A\right) & = & \frac{1}{1+\sum_{x_{i}\neq X}s^{n_{X}-n_{x_{i}}}}. \end{array}$$ (4)

with *s* = *C*′/*C*_0_.

**Fig 6:**
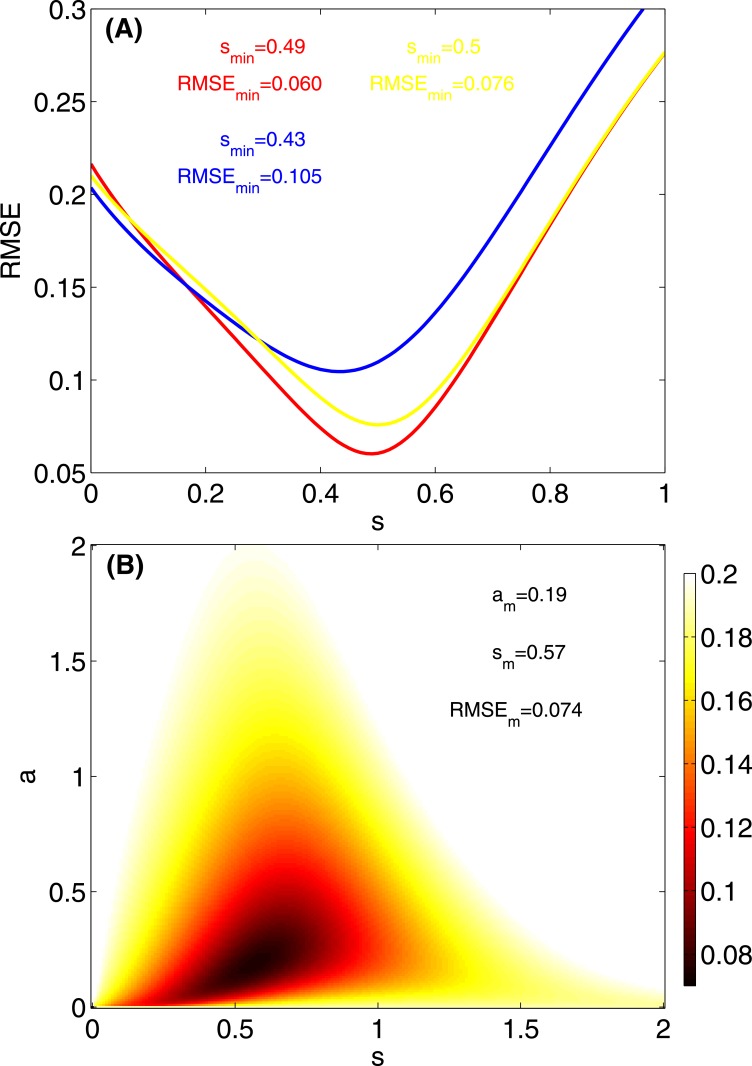
Data fitting. **(A)** Root-mean-square error between the probabilities computed from experimental data (aggregated over all the experiments) and Eq (4). Results for each color are represented by the corresponding colored lines. **(B)** Color-coded root-mean-square error between the probabilities computed from experimental data (aggregated over all the colors across experiments) and Eq (5).

We can estimate the value of the parameter *s* from the experimental data by computing the root-mean-square error (RMSE) of the difference between the probabilities extracted from the experimental data and the probabilities computed using Eq (4). The RMSE, shown in Fig 6A, exhibits a minimum for $s\approx \frac{1}{2}$ indicating that the probability that the signal is of the same value than the current state is twice the probability that the signal has a different value. This result is in agreement with the experimental findings shown in Fig 5B.

We contemplate a second hypothesis where *P*_*pre*_(*x*_*i*_|*A*) is not independent of the current state, so, defining $a=\frac{P_{pre}\left(x_{i}|A\right)}{P_{pre}\left(X|A\right)}$ and *s* = *C*′/*C*_0_, Eq (3) can be now written as

$$\begin{array}{c} P_{post}\left(X|A\right)=\left\{\begin{array}{cc} \frac{1}{1+a\sum_{x_{i}\neq X}s^{n_{X}-n_{x_{i}}}}, & \text{if}\;A=X.\\ \frac{1}{1+a^{-1}s^{n_{X}-n_{A}}+\sum_{x_{i}\neq \left(X,A\right)}s^{n_{X}-n_{x_{i}}}}, & \text{if}\;A\neq X. \end{array}\right. \end{array}$$ (5)

We can now estimate the parameters *a* and *s* by measuring the RMSE between the aggregated probabilities extracted from the experimental data and the probabilities computed from Eq (5). The RMSE exhibits a minimum for the parameters *s* = 0.57 and *a* = 0.19 (Fig 6B). The parameter *a* takes into account non-social information only. For the same number of individuals choosing among the options (i.e., *n*_*x*_1__ = *n*_*x*_2__ = *n*_*x*_3__), *a* = 1 corresponds to equal weight between the options providing a probability of 1/3, as in a random choice. *a* < 1 implies a tendency to remain in your current state since *P*(*X*|*A* = *X*) tends to 1 as *a* tends to zero, while for *a* > 1, individuals tend to change their states more often. The parameter *s* contains the social influence. For *s* = 1, the players’ own opinion and the opinion of the neighborhood are balanced, providing a probability of 1/2 (for *a* = 1). However, when *s* < 1 the opinion of the neighborhood counts more than the players’ own opinion, while the opposite happens for *s* > 1.

### Simulations

**Fig 7:**
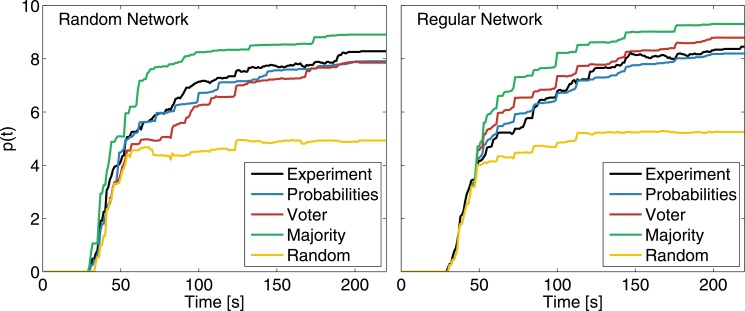
Confronting different models via simulations. Temporal evolution of p(t) for different simulation models in two types of interaction networks. Probabilities correspond to simulations using the experimentally determined probabilities. Experimental data is represented by the black line and it is aggregated over games with the same interaction topology. Simulations are averaged over 50 independent realizations.

We have performed simulations confronting various models with the experimental data, where agents change their state according to the experimentally determined transition probabilities. We have also considered other models with different updating rules. In the Majority rule, agents pick the color of the majority of their neighborhood and ties are broken by random elections among the tied options. In the Voter model, agents pick a color at random from the ones present in the neighborhood. In the Random model however, agents pick a color at random not necessarily present in the neighborhood. The simulations mimic the experimental conditions of the games by using the same number of agents, the same initial conditions, and the same temporal sequence of updates. Fig 7 shows the time evolution of the performance *p*(*t*) for the different models and the experimental data, in both cases aggregated over games with the same interaction topology. In both networks, Majority rule outperformed all other strategies, including the one used by humans. In the random network, Voter performs at the end of the games as well as human strategies. In the regular network, Voter performs slightly worse than Majority, and slightly better than human strategies. For both networks, choosing a color at random independently of the neighbors’ proposals is the worst strategy. Simulations also corroborate that, for the human strategy (Probabilities), there are no differences in the performance of the different networks considered.

## Discussion

We developed a web-based experiment in order to assess how different interaction topologies affect the performance of the aggregation of information and to investigate the underlying decision making process. We found that the behavior of the individuals was well captured by Bayes theory of probability update. Bayesian inference has been observed in human sensorimotor learning [29] and probabilistic cognition [30] as well as in the behavior of many animals [31, 32] (and references therein). The derived expression for the probabilities allows for the estimation of the parameters *s* and *a*, accounting for factors such as the external social influence and the internal preference respectively. Different functional forms for these probabilities are reported in the literature [3, 33–35] for different animals (including humans) and behavioral situations. The parameter fitting to different experimental data shows a high variability in the values of *s* (social influence) and *a* (internal preference), which suggests that they are not only idiosyncratic but also task dependent.

We found that proximity to the source provides a significant advantage in getting the correct answer. The propagation of information can be seen as a wave front emanating from the different sources. The positions shown initially are correctly assigned by the players and almost do not change during the game. Thus, neighbors at distance *d* = 1 will receive this as a constant signal compared to positions that are not known and consequently are changed more often.

With respect to the effect on the aggregation of information of the different interaction topologies considered, we found no significant difference on the performance of the game. This result is in line with the behavior in the context of cooperation. In this case, players have to choose between two options: cooperate or defeat, and they get a payoff according to a payoff matrix and the response of their neighbors. Recent large experiments show no difference in the performance at the level of number of cooperators [20]. However, differences in the performance depending on the network topology are reported in other works. For example, in search problems, efficient networks can outperform inefficient networks [36] and the opposite has also been reported [37, 38]. The reason for this contradictory result is already discussed in reference [36], pointing out that the level of myopia during the search plays a crucial role: in searches performed at intermediate levels, i.e. not too close nor too far, inefficient networks can outperform efficient ones. It also established that humans outperform simulated strategies, pointing out that, for the search class of problems they have considered, agent-based simulations are not sufficiently sophisticated to reflect human responses [37]. In contrast, we found that simulations using agent-based models are useful to find a better performing strategy that solves our problem. By confronting the experimental probabilities with different numerical models based on different decision rules, we found that players did not perform optimally, showing that the Majority rule outperformed players’ outcome for the proposed task. A fundamental question that emerges from this result is why does individuals use Bayesian inference despite the fact that it is not the optimal strategy? Our hypothesis is that Bayesian inference explores and maintains the diversity of options for longer time compared to the Majority rule that quickly narrows the set of final options. A test of this hypothesis could be done in future experiments by decreasing the initial set of positions shown to the players.

The adaptation is an important factor to be considered in games where participants are made to play several times. In our experimental setup we specifically designed the first and the last games to evaluate the adaptation of the players while minimizing the interference with the subsequent games. Players were faster at the end of each experimental session corresponding to a decrease in the inter-proposal times. However, in the games involving human players, we found no differences in the performance of the players. Thus, players familiarized with the game interface during the first game, and no further advantage was obtained from acquiring more experience in the subsequent games.

In all the sessions, the participants approached the correct color code. Whether the games lasted enough time to let the players reach consensus is an open question that needs to be addressed in future experiments. Despite the results reported here, it would be interesting to consider larger networks. We plan to conduct more experiments with larger groups in order to address interaction networks with different neighborhood sizes as well as change the cognitive load of the game by varying the number of positions initially shown and the length of the code. It would also be interesting to study how people form consensus far from the correct solution of the problem, that can be triggered by initializing incompatible targets.

## Supporting Information

S1 Text Supporting text. Detailed description of the phases of the game, the instructions provided to the participants, and the automata game.
(PDF)

S1 Video Activity of the players during one game. From top, the target color code is shown on the first row, second row is left empty (white color) for visualization purposes, and in the following rows the players proposals are displayed. Initially, only the three positions seen by each player are displayed and as the players make their proposals the entire color codes appear.
(MP4)

S1 Fig Temporal evolution of the p-value of two statistical tests. The unpaired Mann-Whitney U test is represented by the black solid line and the paired Wilcoxon signed-rank test corresponds to the red dashed line. At time steps of 1 s, we applied the tests between the distributions of correct answers of the two interaction networks (aggregated over the different games with the same interaction topology). The dotted-dashed line indicates the 5% significance level.
(EPS)

S2 Fig Inter-proposal time for the automata games in each experiment. The mean inter-proposal time for the first (filled symbols) and last game (open symbols), both played against automata, for each experiment. Bars represent the standard error.
(EPS)

S1 Data Experimental data. Data collected during experimental sessions.
(ZIP)
