# Supplementary material for: Collective Intelligence: Aggregation of Information from Neighbors in a Guessing Game
Source: PLoS One. 2016 Apr 19;11(4):e0153586. doi: 10.1371/journal.pone.0153586 (PMC4836688; doi:10.1371/journal.pone.0153586)
Supplement: S1 Text — Detailed description of the phases of the game, the instructions provided to the participants, and the automata game. (PDF) [file pone.0153586.s001.pdf]

Collective intelligence: aggregation of information from neighbors in a guessing game

Toni Pérez[1,*], Jordi Zamora[1], Víctor M. Eguíluz[1]

1 Instituto de Física Interdisciplinar y Sistemas Complejos IFISC (CSIC-UIB). E07122 Palma de Mallorca, Spain.

*** toni@ifisc.uib-csic.es**

Supporting Information

Phases of the game

Each game is divided in 5 phases:

- Phase 1. The goal and instructions of the game are displayed on the screen during 60 seconds.
- Phase 2. During 10 seconds, a different set of positions of the target color code is shown to each player. The positions of the code not shown to the player are displayed as question marks over green background (see Fig. 2 A).
- Phase 3. Each player has to introduce a complete color code, probably using the information provided initially, by filling the ten question marks of the empty color code.
- Phase 4. The player is allowed to perform as many guesses as considered necessary within the game timer countdown (225 s). After introducing a first complete guess, the player is able to see the color codes proposed by the neighbors (see Fig. 2 C).
- Phase 5. Once the timer comes to zero, the game ends and the player is rewarded with a score according to the number of correct colors answered.

After the individual scoring, a historical ranking of all the players with their scores is shown. During this stage the players are synchronized again in order to start the next game simultaneously.

Instructions to the participants

The main goal of the game is shown at the beginning of the experiment as the following itemized list:

- At each game a secret sequence of colors (the Secret Code) will be provided
- Your goal is to find all the colors of the Secret Code before the Timer (top-right) comes to zero
- The more colors you find, the higher will be your Score
- Obtain the highest Score to become the new Leader

At the beginning of each game, the following list of instructions is shown to each participant:

- To begin with, we will show you part of the Secret Code
- We also assigned you a Team (top) that knows parts of the Secret Code
- Color Your Code (bottom) and Update it to see the Codes of your Team
- Look at the Codes of your Team to improve Your Code

A button with a summary of instructions is displayed in the main screen. That summary contained the following instructions:

- Your goal is to find all the colors of the Secret Code before the Timer (top-right) comes to zero
- The more colors you find, the higher will be your Score
- Color Your Code (bottom) as the code initially shown
- You must color all the question marks of Your Code before clicking the Update button
- Look at the Codes of your Team (top) to improve Your Code. They contain parts of the Secret Code

Automata games

In each experimental session, the first and the last game were played against automata. In order to minimize interferences with the subsequent games only two colors per position (red or blue) were used, and five positions of the code were initially shown to the players in these games. The first game was intended to let the players familiarize with the interface of the game. The last game was used to evaluate the adaptation of the players. In these games, no interconnection network was used, each player only interacted with four automata.
